# Dietary and Lifestyle Factors Associated with Self-Esteem in Adolescents: An Exploratory Questionnaire-Based Study

**DOI:** 10.3390/nu18030546

**Published:** 2026-02-06

**Authors:** Andreea Sălcudean, Bianca-Eugenia Osz, Dora-Mihaela Cîmpian, Ramona-Amina Popovici, Cristina-Raluca Bodo, Sarolta Torok, Diana-Mihaela Corodan-Comiati, Raluca Dumache, Andreea-Mihaela Kiș, Mădălina-Gabriela Cincu, Lorena-Mihaela Grebenișan, Elena-Gabriela Strete

**Affiliations:** 1 Department of Bioethics, Deontology and Medical Communication, George Emil Palade University of Medicine, Pharmacy, Science, and Technology of Targu Mures, 540142 Targu Mures, Romania; 2 Faculty of Pharmacy, George Emil Palade University of Medicine, Pharmacy, Science, and Technology of Targu Mures, 540142 Targu Mures, Romania; t_sarolta@yahoo.com; 3 Department of Management and Communication in Dental Medicine, Department I, Faculty of Dental Medicine, Victor Babes University of Medicine and Pharmacy of Timisoara, 300041 Timisoara, Romania; kis.andreea@umft.ro; 4 Psychiatry Clinic 1, County Clinical Hospital Mures, 540142 Targu Mures, Romania; 5 Department of Forensic Medicine, Bioethics, Medical Ethics and Medical Law, Victor Babes University of Medicine and Pharmacy of Timisoara, 300041 Timisoara, Romania; raluca.dumache@umft.ro; 6 Doctoral School of Medicine and Pharmacy, George Emil Palade University of Medicine, Pharmacy, Science, and Technology of Targu Mures, 540142 Targu Mures, Romania; cincu.madalina@yahoo.com; 7 Department of Psychiatry, George Emil Palade University of Medicine, Pharmacy, Science, and Technology of Targu Mures, 540142 Targu Mures, Romaniaelena.buicu@umfst.ro (E.-G.S.)

**Keywords:** adolescents, self-esteem, nutrition, physical activity, lifestyle, BMI

## Abstract

Background: Self-esteem plays a central role in adolescent psychological health and may be shaped by everyday health behaviors such as eating patterns and engagement in physical activity. However, evidence from Eastern European youth remains comparatively limited. Lower levels of self-worth during adolescence have been linked to increased vulnerability to maladaptive behaviors, including substance use. The present study aimed to explore preliminary associations between lifestyle behaviors, nutritional practices, and self-esteem in a sample of Romanian adolescents. Methods: A cross-sectional design was used, involving 113 participants aged 14–18 years. Self-esteem was assessed using the Rosenberg Self-Esteem Scale, while lifestyle behaviors were evaluated through a standardized questionnaire. Body mass index was calculated based on self-reported height and weight. Statistical analyses included Pearson correlation coefficients and multiple linear regression models. Results: Higher self-esteem scores were strongly associated with greater participation in physical activity and adherence to a balanced diet, while inverse relationships were observed with unhealthy dietary habits and higher BMI values. Physical activity emerged as the most influential predictor of self-esteem, accounting for over three-quarters of the variance in Rosenberg scale scores. Conclusions: In this preliminary analysis, physical activity and healthier dietary behaviors were associated with higher self-esteem scores among adolescents. Given the exploratory nature of the study, these findings should be interpreted with caution. They primarily serve to generate hypotheses and highlight the need for future studies with validated instruments, larger samples, and appropriate control for potential confounding factors to better elucidate the relationship between lifestyle behaviors and adolescent self-esteem.

## 1. Introduction

Adolescence is a critical developmental period characterized by significant physical, emotional, and social changes that shape identity, self-worth, and psychosocial adjustment. Typically spanning ages 10 to 19, this stage strongly influences psychological resilience and social functioning. Self-esteem plays a central role during adolescence, affecting how individuals perceive themselves and cope with developmental challenges. Low self-esteem has been consistently linked to adverse mental health outcomes, including depression, anxiety, and social withdrawal, as well as poorer long-term health and socioeconomic prospects [1,2]. Conversely, interventions that support self-esteem development may enhance resilience and promote more adaptive coping strategies during this formative period [2,3].

The formation of self-esteem occurs within a complex network of social influences rather than in isolation. Experiences of stigmatization, exclusion, or unfavorable evaluation in educational or community environments may erode adolescents’ sense of self-worth and contribute to problematic developmental outcomes [4]. Conversely, warm and supportive relationships with parents are linked to stronger self-esteem and more successful psychosocial adjustment [5]. At a broader level, self-esteem is closely connected to emotional regulation and levels of psychological strain during adolescence [6]. Evidence from longitudinal studies indicates that self-esteem typically increases progressively from early adolescence into adulthood [7] and shows positive associations with both academic achievement and overall life satisfaction [8]. Collectively, these findings emphasize self-esteem as a key marker of healthy psychosocial development in adolescence.

Extensive research has demonstrated that diminished self-esteem is reliably associated with elevated depressive and anxiety symptoms, as well as impaired social functioning. Evidence from both cross-sectional and longitudinal investigations identifies low self-esteem as a significant vulnerability factor for the development of depression and anxiety. Moreover, self-esteem predicts the later emergence of clinically relevant internalizing symptoms across diverse populations, even when the influence of stressful life events is considered [9,10,11,12,13,14].

Low self-esteem not only amplifies the psychological impact of stress but may also independently contribute to depressive symptoms, with evidence suggesting a bidirectional relationship that reinforces emotional distress [10]. Adolescents with low self-esteem are more prone to social withdrawal and heightened sensitivity to negative social evaluation, increasing vulnerability to both depression and anxiety, particularly in interpersonal contexts [10,15]. The frequent co-occurrence of social anxiety and depressive symptoms highlights the role of self-evaluative processes in shaping emotional outcomes, while contextual factors such as family and school environments may either exacerbate or buffer these associations [16,17].

From a biopsychosocial perspective, modifiable lifestyle behaviors—including physical activity, diet, and body weight—are increasingly recognized as contributors to adolescent emotional well-being and self-esteem. Physical activity has been consistently linked to higher self-esteem, supporting the idea that health behaviors influence self-perceptions and mental health trajectories [18]. Similarly, evidence across multiple lifestyle domains indicates that nutrition, sleep quality, and physical activity are associated with better quality of life and more positive self-evaluations in adolescents [19]. Diets rich in fruits, vegetables, and other nutrient-dense foods have also been associated with more favorable psychological profiles and healthier body image, factors closely related to emotional regulation and cognitive functioning [20,21].

Physical activity is consistently associated with better mental well-being in adolescents and students, including higher subjective well-being, improved emotional regulation, and reduced stress and anxiety [22,23,24,25,26,27,28]. Beyond physical health benefits, regular exercise also supports positive body image and psychological resilience, particularly in academically demanding contexts [22,23,24,25,29,30].

The interplay between diet, physical activity, body image, and mental health is complex and influenced by environmental, psychosocial, and sociodemographic factors. Evidence suggests that food environments, lifestyle patterns, and academic stress collectively shape eating behaviors, body dissatisfaction, and overall well-being during adolescence [31,32,33,34,35]. Within this context, lifestyle behaviors may be understood as modifiable factors linked to adolescents’ self-perceptions and emotional health.

Prior studies have established links between self-esteem and selected lifestyle behaviors, most notably physical activity and certain nutritional patterns [18,19,20,21]. However, much of the existing literature considers these behaviors separately, focuses largely on Western populations, or relies on samples with limited cultural heterogeneity. Consequently, less is known about the combined contribution of dietary quality (balanced versus unhealthy eating), physical activity, and body mass index to self-esteem when examined simultaneously within adolescent samples. Research involving adolescents from Eastern Europe, including Romania, remains relatively scarce compared with that conducted in Western countries, despite evidence that cultural norms, socioeconomic conditions, and public health contexts may uniquely influence both lifestyle behaviors and self-evaluative processes in this region [31,32,33,34,35].

Romanian adolescents constitute a particularly relevant population for investigation, considering recent trends observed across Central and Eastern Europe, such as shifts toward less healthy dietary patterns, increased sedentary behavior, and rising prevalence of overweight and obesity. Although interest in the psychological correlates of lifestyle behaviors in this region has grown, such outcomes remain underrepresented in the literature relative to Western contexts. Generating empirical data from Romanian adolescents can therefore provide culturally informed evidence and facilitate meaningful comparisons with findings from other European settings.

Guided by biopsychosocial and lifestyle medicine frameworks, the present study conceptualizes self-esteem as an outcome influenced by an interconnected set of modifiable behaviors, including physical activity, dietary habits, and weight status. Building on previous research, this study aimed to examine the relationships between self-esteem and dietary patterns (balanced and unhealthy nutrition), physical activity and supplement use, and BMI in a sample of Romanian adolescents. By analyzing these lifestyle factors concurrently, the study seeks to offer a more comprehensive understanding of how everyday health behaviors relate to adolescents’ perceptions of self-worth.

## 2. Materials and Methods

### 2.1. Study Design, Participants, and Data Collection

The research was conducted using a cross-sectional, observational approach. The sample consisted of 113 adolescents selected through convenience sampling from four state secondary schools in Târgu Mureș, Romania, as well as nearby rural areas. School participation was based on ease of access and formal approval to collaborate in the study. Students enrolled in grades 9 through 12, corresponding to an age range of 14 to 18 years, were invited to participate. Eligibility criteria included being within the specified age range, current attendance at one of the participating schools, and receipt of informed consent from a parent or legal guardian. Participants were excluded if their questionnaires were incomplete or if they reported chronic physical or mental health conditions that might affect lifestyle-related behaviors.

Participants were recruited via school-wide announcements and with the assistance of classroom teachers. Detailed study information and consent forms for parents or legal guardians were provided approximately one week prior to the survey administration. Participation was voluntary, anonymous, and uncompensated. The questionnaire was delivered in Romanian and was linguistically adapted to ensure comprehensibility and cultural appropriateness for adolescent respondents.

Data were collected between April and May 2025. Questionnaires were completed online using Google Forms and administered in classroom environments under teacher supervision to ensure consistent administration procedures and minimal disruption to instructional activities. Of approximately 150 eligible students, 113 provided complete responses, yielding a response rate of 75.3%. The resulting sample had a mean age of 16.98 years (SD = 1.28) and consisted of 68 girls (60.2%) and 45 boys (39.8%).

To assess whether the sample size was adequate, a post hoc power analysis was performed using G*Power version 3.1. Assuming a two-tailed correlation test with a significance level of α = 0.05, a moderate observed effect size (r = 0.32), and a sample size of 113, the calculated statistical power (1–β) was 0.85, indicating sufficient sensitivity to detect moderate relationships between lifestyle variables and self-esteem.

Ethical clearance for the study was granted by the Scientific Research Ethics Committee of the University of Medicine, Pharmacy, Science and Technology of Târgu Mureș, Romania (approval number 3678/30 April 2025).

### 2.2. Measures

#### 2.2.1. Self-Esteem

Self-esteem was measured using the 10-item Rosenberg Self-Esteem Scale (RSES), applied in its Romanian-validated form. Respondents evaluated each statement using a 4-point Likert-type scale ranging from 1 (“Does not describe me at all”) to 4 (“Describes me completely”). Negatively worded items (items 4 and 8) were reverse-coded prior to analysis, and a total self-esteem score was calculated by summing responses across all items, with higher scores reflecting greater self-esteem. Only questionnaires with complete responses to all 10 RSES items were included in self-esteem analyses; cases with missing item data were excluded.

The scale demonstrated strong internal reliability within the current sample, with a Cronbach’s alpha coefficient of 0.88, exceeding the commonly accepted threshold of 0.70 for research use in behavioral and psychological studies.

#### 2.2.2. Healthy Lifestyle and Mental Health Questionnaire

Lifestyle behaviors were assessed using the Healthy Lifestyle and Mental Health Questionnaire, developed specifically for this study in Romanian adolescents. Item development was informed by components of established school-based health surveys, particularly the WHO Global School-based Student Health Survey (GSHS) and the Health Behavior in School-aged Children (HBSC) study, which provide standardized and psychometrically supported items on physical activity, dietary behaviors, and mental health in adolescents [36,37]. The initial item pool was adapted to the Romanian context to ensure age-appropriate language and cultural relevance. Content validity was evaluated by a panel of three experts in adolescent health and psychology, who reviewed each item for relevance and coverage of the target domains. A pilot administration with 10 students (not included in the final sample) was then conducted to assess clarity, acceptability, and completion time; no substantial wording changes were required after this pilot.

1. Physical Activity Scale (3 items)

This section evaluated engagement in physical activity by assessing frequency, duration, and intensity of exercise or sports participation. Responses were recorded on a 5-point Likert scale ranging from 1 (“never”) to 5 (“daily”). A composite Physical Activity Index was calculated by summing responses across the three items, with higher scores representing greater levels of physical activity. The scale demonstrated good internal reliability in the present sample (Cronbach’s α = 0.83).

2. Nutrition Scale (14 items)

Dietary behaviors were assessed through items capturing a wide range of food choices and consumption habits. All items were rated on a 5-point Likert scale (1 = “never,” 5 = “daily”). Guided by theoretical frameworks in adolescent nutrition research, two distinct dietary indices were constructed:

- Healthy Nutrition Index (7 items):

This index included items reflecting positive dietary behaviors, such as regular intake of fruits, vegetables, lean protein sources, dairy products, and meals prepared at home.

- Unhealthy Nutrition Index (7 items):

This index captured the frequency of consuming foods considered nutritionally poor, including sweets, fast food, sugar-sweetened beverages, packaged snacks, and other energy-dense items.

Scores for each index were obtained by summing item responses within the respective subscale. Higher values on the Healthy Nutrition Index indicated more favorable eating habits, whereas higher scores on the Unhealthy Nutrition Index reflected more frequent unhealthy dietary practices. Internal consistency was acceptable to good for both subscales (Healthy Nutrition: α = 0.81; Unhealthy Nutrition: α = 0.78).

3. Mental Health and Body Image Scale (10 items)

This component assessed adolescents’ perceptions of body image, self-acceptance, social influences, satisfaction with body weight, and the perceived emotional impact of nutrition. Although included to provide a broader assessment of health-related perceptions, this scale was not incorporated into the current analyses, as it did not directly correspond to the study’s primary objective of examining lifestyle factors associated with self-esteem. The scale showed good internal consistency (Cronbach’s α = 0.84).

In addition to the domain-specific indices, a total lifestyle score was computed by summing the items from the physical activity and nutrition subscales, so that higher scores reflected more frequent engagement in physical activity, more regular consumption of healthy foods, and less frequent intake of unhealthy foods and beverages. For descriptive classification purposes, a cut-off score of 30 was derived from the distribution of total lifestyle scores (corresponding approximately to the lower tertile). Participants scoring below this threshold were categorized as having an ‘unbalanced’ lifestyle, whereas those scoring at or above 30 were categorized as having a more ‘balanced’ lifestyle pattern.

The lifestyle questionnaire also included one item assessing the frequency of nutritional supplement use (e.g., vitamins, minerals, protein supplements).

Previous research indicates that, in adolescence, dietary supplement use is closely linked to sport participation and physically active lifestyles, often driven by performance-, recovery-, or physique-related motives [38]. In line with this, we constructed an exploratory composite indicator by combining the three physical-activity items with the supplement-use item, labelled “Physical Activity & Supplements”. This composite was intended to capture a broader activity- and performance-oriented lifestyle profile and was included as an additional lifestyle index in correlation and regression analyses, alongside the separate nutrition indices and BMI. Because it aggregates related but non-identical behaviors, and supplement use was assessed with a single item, the “Physical Activity & Supplements” index is interpreted cautiously. All 113 adolescents included in the analyses completed all items of the lifestyle questionnaire; therefore, no missing data imputation was required for these scales, and partial questionnaires were excluded from the final sample.

A concise overview of the questionnaire structure, scoring procedures, descriptive statistics, and reliability coefficients for each domain is presented in Table 1.

- Body Mass Index (BMI)

Body Mass Index (BMI; kg/m^2^) was calculated using self-reported height and weight. BMI values were inspected for plausibility (range and distribution), and no extreme or implausible values were identified; therefore, all cases were retained in the analyses. For descriptive purposes, participants were categorized as underweight (BMI < 18.5 kg/m^2^), normal weight (18.5–24.9 kg/m^2^), overweight (25.0–29.9 kg/m^2^), or obese (≥30.0 kg/m^2^), according to World Health Organization adult criteria. In the main statistical analyses, BMI was treated as a continuous variable.

### 2.3. Statistical Analysis

All analyses were conducted using IBM SPSS Statistics (version 25). Statistical significance was set at α = 0.05, and all tests were two-tailed. Descriptive statistics (means, standard deviations, and frequencies) were computed for all variables. Associations between lifestyle indices and self-esteem were examined using Pearson correlation coefficients, which provide estimates of the strength and direction of linear relationships between variables. Gender differences were assessed using independent-samples *t*-tests.

All analyses were performed on complete cases. The final analytic sample included 113 adolescents who provided complete data on the lifestyle questionnaire, self-reported height and weight, and Rosenberg self-esteem scores; questionnaires with missing lifestyle items or incomplete RSES responses were excluded from the analyses, and no missing data imputation was applied. For the multiple linear regression model, standard assumptions were examined. Visual inspection of histograms and Q–Q plots of standardized residuals suggested approximate normality, and plots of residuals versus fitted values indicated no marked violations of linearity or homoscedasticity. Multicollinearity was assessed using variance inflation factors, which were all below 2, indicating no problematic multicollinearity among the variables included in the analysis.

Given the exploratory nature of the study and the relatively small sample size, advanced analyses were not applied, as statistical power would have been insufficient for reliable estimation of complex models. Instead, the analytic strategy focused on identifying preliminary patterns of association, which can inform the design of future research with larger and more diverse samples.

No participant attrition occurred, as data were collected at a single time point. Missing data were minimal and were handled using complete-case analysis. Given the low proportion of missing values, this approach was considered unlikely to substantially bias the results.

## 3. Results

### 3.1. Sample Characteristics

The final sample included 113 adolescents (60.2% female; mean age 16.98 ± 1.28 years). Mean weight was 66.20 ± 15.80 kg and mean height 165.80 ± 7.49 cm. Around 53% lived in rural areas, 46% in urban areas. The rural or urban status was determined based on the geographical location of the school each participant attended.

The sample included 113 Romanian adolescents (45 male and 68 female) aged between 14 and 18 years. The distribution was dominated by participants aged 18 years (*n* = 59; 52.3%), followed by those aged 16 years (*n* = 18; 15.9%), 17 years (*n* = 17; 15.0%), 15 years (*n* = 11; 9.7%), and 14 years (*n* = 7; 6.2%). The gender distribution across age groups is presented in Table 2.

Although body mass naturally fluctuates during adolescence, participants self-reported their current weight and height, based on which the body mass index (BMI) was calculated. The average BMI in the sample was 23.8 (SD = 2.9), indicating that most participants were within the normal weight range. Specifically, 63.8% of adolescents had a BMI between 18.5 and 24.9 (normal), while 24.8% were categorized as overweight, 4.8% as obese, and 6.6% as underweight. This distribution served as a reference point for analyzing associations between weight status, self-esteem, and body image perception.

When asked about their ideal body weight, female participants tended to report lower ideal weights compared to their current values, whereas males generally reported ideal weights equal to or slightly above their current weight. Notably, 60.3% of girls expressed concerns about their weight and reported a desire to lose weight, compared to 46.7% of boys. These gender-based differences support previous findings that adolescent girls are more likely to internalize societal pressure regarding thinness and appearance.

Regarding dietary patterns, most participants (*n* = 67; 59.3%) reported not following any specific diet, whereas approximately one-fifth adhered to a balanced diet (*n* = 25; 22.1%). Smaller subgroups followed low-carbohydrate (*n* = 8; 7.1%), portion-reduction (*n* = 8; 7.1%), or gluten-free diets (*n* = 5; 4.4%), and no participant reported following a vegetarian diet (*n* = 0; 0.0%). These diet types were self-identified by participants in response to a single item asking them to choose the option that best described their current eating behavior (see Table 2 for a full breakdown of dietary pattern distribution).

The total lifestyle score, calculated by summing responses from the physical activity and nutrition subscales, provides an overall index of health-related behaviors. In this sample, the mean lifestyle score was 31.9 (SD = 6.2), suggesting a moderate level of adherence to healthy habits. To classify participants’ lifestyle patterns, a cut-off score of 30 was established based on the lower tertile of the score distribution. Using this threshold, 88.5% of adolescents were categorized as leading an “unbalanced” lifestyle, characterized by low physical activity, insufficient consumption of healthy foods, and frequent intake of unhealthy items.

### 3.2. Lifestyle Determinants of Self-Esteem

Before exploring the relationships between variables, descriptive statistics for self-esteem and lifestyle indices were computed to provide an overview of the sample’s characteristics (Table 3). Overall, participants reported moderate levels of physical activity, generally balanced nutritional habits, and adequate self-esteem, with variability across individual indicators.

**Figure 1:**
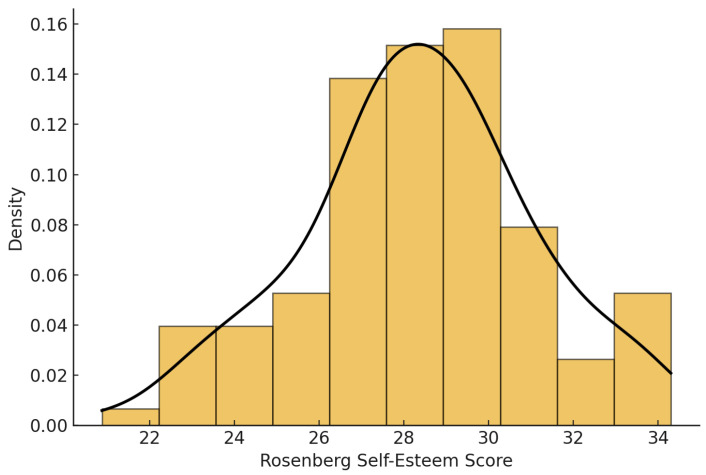
Distribution of Rosenberg Self-Esteem Scale total scores among Romanian adolescents (*n* = 113). The histogram indicates a normal distribution (Mean = 28.68, SD = 2.98), suggesting adequate variability for correlation and regression analyses.

Before examining the relationships among the main study variables, we assessed the distribution of self-esteem scores to ensure adequate variability and normality for subsequent analyses. Figure 1 illustrates the distribution of Rosenberg Self-Esteem Scale total scores in the sample, showing a near-normal shape and confirming the suitability of the data for parametric tests.

To explore the associations between lifestyle behaviors, BMI, and self-esteem, Pearson correlation analyses were conducted. Table 4 displays the correlations between self-esteem and lifestyle variables, indicating significant positive associations of self-esteem with balanced nutrition (r = 0.469, *p* < 0.001) and the Physical Activity & Supplements composite index (r = 0.836, *p* < 0.001), and negative associations with unhealthy nutrition (r = –0.334, *p* < 0.001) and BMI (r = –0.646, *p* < 0.001). Mean scores were 11.4 (SD = 3.2) for healthy nutrition, 9.3 (SD = 2.9) for unhealthy nutrition, and 11.7 (SD = 3.1) for the Physical Activity & Supplements composite index. The total lifestyle score had a mean of 31.9 (SD = 6.2), indicating a moderately favorable lifestyle pattern at the group level. As shown in Table 4, several lifestyle indices demonstrated significant correlations with self-esteem, suggesting that adolescents with healthier lifestyle profiles generally reported greater self-esteem.

To identify the lifestyle determinants of self-esteem, a multiple linear regression analysis was conducted. In the multiple regression analysis (Table 5), physical activity and balanced nutrition remained significant variables linked to self-esteem after controlling for BMI. The lifestyle variables, such as diet quality and physical activity, emerged as significant factors that may contribute to self-esteem, explaining a meaningful proportion of the variance in adolescents’ self-esteem scores.

**Figure 2:**
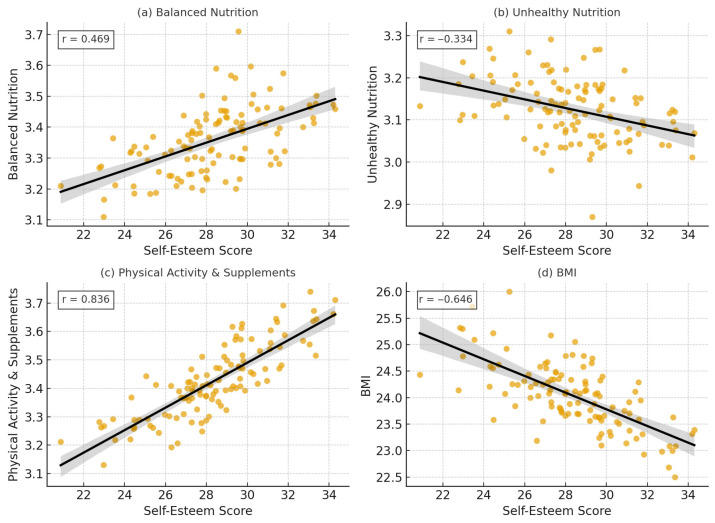
Scatter plots illustrating bivariate associations between self-esteem and (**a**) balanced nutrition, (**b**) unhealthy nutrition, (**c**) physical activity and supplements, and (**d**) body mass index (BMI). Pearson correlation coefficients (r) and *p*-values are shown in each panel. Self-esteem was positively related to balanced nutrition and physical activity and negatively related to unhealthy nutrition and BMI.

To further illustrate the patterns identified in the correlation and regression analyses, Figure 2 presents scatter plots depicting the bivariate associations between self-esteem and each lifestyle indicator. The graphical distributions confirm the linear trends observed in the statistical results: higher levels of balanced nutrition and physical activity were associated with higher self-esteem, whereas greater unhealthy nutritional behaviors and higher BMI were related to lower self-esteem. The visual plots add clarity regarding the spread and consistency of these associations across individual participants.

**Figure 3:**
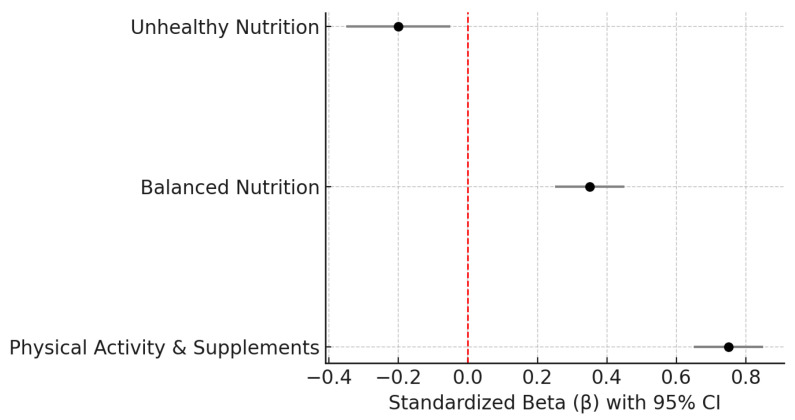
Standardized regression coefficients (β) and 95% confidence intervals for factors associated with self-esteem in Romanian adolescents. Physical activity showed the strongest positive association with self-esteem, followed by balanced nutrition, whereas unhealthy nutrition was negatively associated.

To clarify the relative strength of each predictor within the multivariable model, Figure 3 displays the standardized regression coefficients and their confidence intervals. Physical activity showed the strongest positive contribution to self-esteem, followed by balanced nutrition, whereas unhealthy nutrition exhibited a moderate negative association. These coefficients align with the bivariate patterns and underscore the central role of physical activity in adolescents’ self-evaluations.

### 3.3. Gender Differences in Self-Esteem

Girls reported significantly higher self-esteem than boys, t(111) = 2.14, *p* = 0.035.

**Figure 4:**
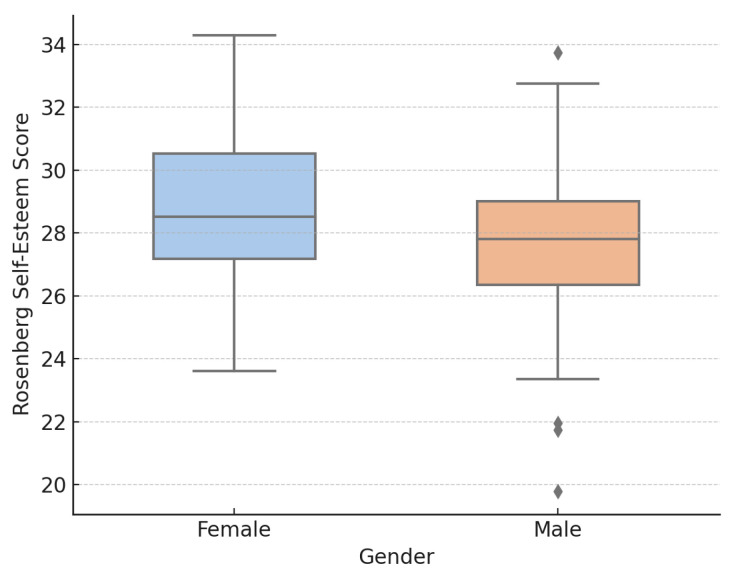
Gender differences in Rosenberg Self-Esteem Scale total scores. Female adolescents reported significantly higher self-esteem than males (t(111) = 2.14, *p* = 0.035). Boxes represent interquartile ranges (IQR), horizontal lines indicate medians, whiskers extend to 1.5 × IQR, and diamonds represent outliers.

Gender differences in self-esteem were also examined. As shown in Figure 4, female adolescents reported significantly higher self-esteem than males. Although the effect size was modest, the distributional differences illustrated in the boxplot are consistent with established gender patterns in adolescent self-perception.

## 4. Discussion

The present study examined potential associations between lifestyle factors—namely physical activity, balanced and unhealthy nutrition, and body mass index (BMI)—and self-esteem among Romanian adolescents. The results suggested that healthier lifestyle patterns, particularly higher levels of physical activity and balanced dietary habits, tended to be positively associated with self-esteem, whereas higher BMI and unhealthy eating behaviors were associated with lower self-esteem. Although the multiple regression model explained a substantial proportion of the variance in self-esteem (R^2^ = 0.773), this value should be interpreted with caution. The high R^2^ likely reflects the conceptual overlap among lifestyle indicators and the strong internal coherence of health-related behaviors in adolescence. Because the present study used a cross-sectional design and correlated lifestyle indices, the predictive strength observed here should be viewed as exploratory rather than definitive. To avoid overinterpretation, the associations reported in this study should be understood strictly as correlational. The cross-sectional design, the reliance on self-report measures, and the conceptual overlap among lifestyle indicators preclude causal inferences. The findings, therefore, reflect patterns of co-occurrence rather than directional mechanisms. Consequently, we cannot determine whether healthier lifestyle behaviors lead to higher self-esteem, whether adolescents with higher self-esteem are more likely to adopt healthier behaviors, or whether both are shaped by unmeasured contextual factors (e.g., family environment, socioeconomic status, or underlying mental health symptoms). Nevertheless, the finding suggests that lifestyle components may exert a cumulative influence on adolescents’ psychological adjustment. Future longitudinal studies with larger samples are needed to verify these combined effects and to further disentangle potential collinearity among associated factors.

The strongest predictor of self-esteem was physical activity, confirming the extensive evidence that engagement in regular exercise contributes to positive self-perception, body satisfaction, and emotional stability during adolescence. Physical activity enhances self-efficacy, promotes mastery experiences, and provides opportunities for social inclusion and peer recognition, all of which are core components of self-esteem development. Neurobiological research further supports these findings by demonstrating that exercise induces neurochemical changes, such as increased endorphin release and modulation of stress-related hormones, resulting in improved mood and self-esteem. The pronounced relationship observed in this study (r = 0.836, *p* < 0.001) suggests that physical activity may represent not only a behavioral correlate but also a psychological mechanism that supports adolescents’ resilience and self-concept formation. These findings are consistent with the regression coefficients illustrated in Figure 3, which visually clarify the relative magnitude of each predictor while reinforcing the central role of physical activity in self-esteem. In addition, the combined indicator of physical activity and supplement use reflects a broader health-oriented behavioral profile, consistent with evidence that adolescents who engage in regular physical activity often adopt complementary practices aimed at optimizing well-being. Regular physical activity has been shown to improve body satisfaction, emotional regulation, and mental well-being. At the same time, sedentary behavior tends to undermine mental health and increase the risk of anxiety [22]. This is also highlighted by larger research that identifies sedentary behavior as a risk factor for anxiety and stress in young people and adults [23,24,25,26]. While the present findings align with widely reported benefits of physical activity, the effect sizes observed here may also reflect unmeasured variables, such as family environment, peer dynamics, or school context, which were not controlled for in the current design.

Balanced nutrition was positively associated with self-esteem, with adolescents reporting healthier eating patterns also reporting higher life satisfaction. Diet quality, particularly higher intake of fruits, vegetables, and whole grains, has been linked to better cognitive performance, emotional regulation, and lower depressive and anxiety symptoms. Conversely, higher consumption of processed foods, snacks, and sugary drinks was negatively associated with self-esteem, potentially reflecting body dissatisfaction and negative affect. These findings align with existing evidence that healthier dietary patterns support more positive self-perceptions and psychological well-being in adolescents. Scientific evidence underlies this claim, linking healthy dietary patterns to greater body appreciation and a reduced risk of psychopathology, especially among adolescents [20,21,35], with support for specific components, such as fruit/vegetable consumption and regular meals [34,39]. Furthermore, diets high in processed foods and sugary drinks are associated with stress and negative body image states among college students, although some reports find null results for ultra-processed foods on body image measures in some cohorts. The converging message is that unhealthier eating patterns may accompany increased stress and poorer body image satisfaction [20,32,35,40,41].

BMI was negatively correlated with self-esteem, a finding consistent with prior studies demonstrating that higher body weight is often accompanied by lower perceived self-worth among adolescents. The relationship between diet and body image is not universally linear, with some studies reporting null or mixed associations between ultra-processed food consumption and body image dissatisfaction, highlighting the complexity and potential moderating factors (e.g., socio-cultural context, measurement approaches) on how levels of dietary processing are reflected in body image self-esteem [39]. Other studies highlight associations between processed or sugary foods and adverse body image and stress outcomes, as evidenced by links between sugary drinks, low fruit consumption, and stress or negative body image states among adolescents [20,32,34]. However, the literature contains divergent findings regarding this statement in some cohorts [35,39]. This relationship can be understood through both social and psychological pathways. Nonetheless, these interpretations should be considered cautiously, as BMI is influenced by multiple sociocultural and developmental factors that were not fully captured in this study. Socially, adolescents with higher BMI may experience stigma, teasing, or internalized weight bias, all of which undermine body image and confidence. Psychologically, higher BMI may be associated with increased preoccupation with physical appearance and reduced satisfaction with body functionality. Prior studies have demonstrated that higher body weight is often accompanied by lower perceived self-worth among adolescents. This relationship can be understood through both social and psychological pathways: adolescents with higher BMI may experience stigma, teasing, or internalized weight bias, all of which undermine body image and confidence, and may also become more preoccupied with physical appearance and less satisfied with body functionality. However, these interpretations should be considered cautiously, as BMI is influenced by multiple sociocultural and developmental factors that were not fully captured in this study. Furthermore, systematic reviews and meta-analyses conducted among adolescents and young adults show positive associations between sedentary time and anxiety and broader mental health concerns, with moderate evidence for increased risk of anxiety related to more sitting time, although findings may vary by domain and measurement [24,26]. Longitudinal and cross-sectional studies highlight that sedentary lifestyles are associated with higher perceived stress and poorer mental health in some populations, highlighting the health risks of inactivity beyond caloric balance or physical fitness [22,25,26]. Among adolescent students, context-specific factors, such as academic stress and campus life, that influence activity levels and associated mental health outcomes reinforce the value of integrating physical activity promotion into the university environment to support mood and well-being [22,32,33]. Given the associations between self-esteem and lifestyle factors, integrating the promotion of physical activity, sleep, and stress management into school and community programs may indirectly improve self-esteem and therefore reduce the risk of depression and anxiety [18,19,42]. Converging evidence indicates that factors related to lifestyle, physical activity, sleep, diet, stress exposure, and broader social and environmental contexts contribute significantly to increasing self-esteem in adolescents and young adults. Given the link between self-esteem and depression/anxiety, identifying and modifying these lifestyle factors represents a viable public health strategy for improving mental health outcomes at the population level [9,10,11,18,19,41,42].

The gender analysis indicated that female adolescents scored significantly higher on self-esteem than males, although the effect size was modest. This result contrasts with some Western findings where boys typically report higher self-esteem, yet it is not unprecedented. Cultural context may partly explain this pattern, as Romanian adolescent girls might benefit from recent social shifts emphasizing self-expression, personal development, and social support in school environments, factors that may buffer against the traditionally lower self-esteem reported in females. Given the localized nature of the sample and the convenience-based recruitment, these cultural explanations remain speculative and require replication in larger, more diverse Romanian cohorts. Alternatively, boys may face different sociocultural expectations regarding performance and independence, potentially heightening stress and self-criticism during this developmental period. Family atmosphere and social contexts affect self-esteem and, in turn, influence negative emotional states. For example, family environment may affect self-esteem, which then mediates relationships with negative emotions, such as anxiety and depression in adolescents [41]. This indicates that lifestyle and contextual factors (e.g., family functioning, social support) are levers for action to improve self-esteem and mental health at the population level. School climate and family environments emerge as important contextual determinants of how self-esteem translates into mental health outcomes; interventions that enhance peer support networks, teacher support, and positive family functioning may mitigate the negative impact of low self-esteem on social integration and internalizing symptoms [15,17,41].

The findings support multidimensional models of adolescent self-esteem that consider behavioral, physical, and psychosocial factors. They provide preliminary evidence from an Eastern European context, which is underrepresented in research on adolescent psychological and nutritional health. Associations between lifestyle behaviors and self-esteem highlight the interconnection of physical and mental well-being, suggesting that promoting healthy habits may support psychological resilience. Practically, these results underscore the potential value of school-based programs combining physical activity and nutrition education, as well as fostering environments that emphasize self-acceptance, body functionality, and health-oriented goals. Family and community involvement may further reinforce these behaviors through modeling and support, although causal effects cannot be inferred from the current data. In the field of public health, there is increasing emphasis on identifying lifestyle-related contributors to self-esteem to support prevention and intervention efforts. The assertion that low self-esteem is associated with depression, anxiety, and social integration problems is supported by systematic evidence confirming the link between low self-esteem and depression/anxiety, strengthening the generalizability of this association across age groups [11,12]. Furthermore, research conducted on adolescents identifies low self-esteem as a risk factor for the development of depression, reinforcing the broad relevance of this association throughout adult life [42]. Low self-esteem is also associated with poorer social functioning and greater social-evaluative preoccupation. It has been argued that low self-esteem is linked to greater social anxiety and more negative social experiences, which in turn may hinder social integration and support networks [15]. Together, these sources support the main claim that low self-esteem is associated with depression, anxiety, and social integration difficulties across all age groups and settings, with converging evidence from longitudinal studies, meta-analyses, and cross-sectional studies [9,10,11,12,15,16,17,41,43].

Reports from cross-cultural and student studies further underscore the public health imperative to identify lifestyle-related factors that contribute to self-esteem. For example, research in diverse student populations shows that dysfunctional attitudes and low self-esteem are risk factors for depression and anxiety, suggesting that culturally and developmentally appropriate psychosocial and lifestyle interventions could help reduce internalizing symptoms [11,12,42]. Furthermore, the relationship between self-esteem and internalizing symptoms is not fixed across environments; for example, school climate may moderate the impact of self-esteem on internalizing problems, suggesting that favorable contexts may mitigate the potential negative effects of low self-esteem [17]. Public health programs aimed at reducing internalizing disorders should consider screening for low self-esteem as an indicator of risk and include components that strengthen self-esteem through evidence-based approaches (e.g., resilience building, social skills training, or cognitive-behavioral elements that address negative self-views) in adolescents and young adults in the developing world [9,11,42].

While the cross-sectional design precludes causal conclusions, the high proportion of explained variance suggests that the measured lifestyle behaviors are meaningful contributors to adolescents’ self-esteem. Future longitudinal and interventional studies are warranted to examine the directionality and potential mediating mechanisms, such as body image, emotional regulation, or perceived social support. Additionally, integrating objective measures of physical activity (e.g., accelerometers) and dietary intake could enhance the accuracy and generalizability of findings.

Overall, the present study suggests that self-esteem in adolescence may be influenced by modifiable lifestyle behaviors. Physical activity, balanced nutrition, and healthy body image perceptions appear to work synergistically in promoting adolescents’ self-worth. These results emphasize the need for integrated, multidisciplinary strategies, spanning schools, families, and community health programs, to nurture both the physical and psychological development of young people. Comprehensive studies that address both individual and contextual factors influencing self-esteem can form the basis of effective strategies to improve psychosocial outcomes for adolescents.

### Strengths and Limitations

A key strength of this study is its integrated examination of multiple lifestyle dimensions, physical activity, balanced and unhealthy dietary patterns, and body mass index, within a single analytical framework. This multidimensional approach allows for a more comprehensive view of how everyday health behaviors relate to adolescents’ self-esteem. The use of a well-established measure of self-esteem (the Rosenberg Self-Esteem Scale) and internally consistent lifestyle indices supports acceptable measurement reliability. However, the Healthy Lifestyle and Mental Health Questionnaire is a newly developed instrument and, despite expert review, pilot testing, and satisfactory internal consistency, requires further psychometric validation in larger and more diverse samples. Accordingly, the lifestyle indices should be considered preliminary. The present study represents an initial step toward hypothesis generation and instrument refinement, and future research with larger samples will be necessary to complete formal validation procedures.

An additional contribution lies in the study’s focus on a Central and Eastern European context that is underrepresented in adolescent health research. By examining Romanian adolescents, the findings provide exploratory, culturally specific evidence on associations between lifestyle behaviors and self-esteem and offer a basis for comparison with studies from other regions.

Several limitations should be acknowledged.

Analyses were necessarily limited to descriptive statistics, bivariate correlations, and a parsimonious regression model, without adjustment for key confounders such as socioeconomic status, mental health symptoms, sleep, or family and social factors. Moreover, the brief questionnaire format may not have fully captured the complexity of physical activity and dietary behaviors. As such, the findings should be viewed as exploratory and interpreted with caution, underscoring the need for larger, methodologically robust studies to confirm and extend these results.

A key limitation of this study is the lack of temporal direction inherent in its cross-sectional design. Because all variables were measured at a single time point, causal relationships and the direction of observed associations cannot be determined. For instance, higher self-esteem may promote greater physical activity, physical activity may enhance self-esteem, or both may be influenced by unmeasured factors. Therefore, findings should be interpreted as associative rather than causal.

The small, convenience-based sample drawn from a single urban area limits statistical power and generalizability. The cross-sectional design precludes causal inference, and reliance on self-reported measures introduces potential recall and social desirability biases, particularly for diet, physical activity, and BMI.

As the primary aim of this study was exploratory in nature, no a priori sample size calculation was performed. Participation in the study was voluntary, which may have introduced self-selection bias. Adolescents with greater interest in health-related topics or higher engagement in physical activity may have been more likely to participate, potentially leading to an overrepresentation of healthier behaviors. This may have resulted in an overestimation of favorable health indicators and should be considered when interpreting the findings.

Regarding representativeness, the sample was drawn from a specific geographic area and school setting, which may limit the generalizability of the results. While the findings are relevant to adolescents in similar contexts, caution is warranted when extrapolating results to other regions of Romania or to adolescents in different cultural or socioeconomic environments.

Self-esteem, physical activity, and dietary behaviors were assessed using self-reported measures, which are susceptible to recall bias and social desirability bias. Participants may have overreported socially desirable behaviors (e.g., physical activity) or underreported unfavorable ones (e.g., unhealthy dietary patterns), potentially leading to misclassification. Such biases are likely to be non-differential and may have attenuated observed associations.

Covariates were selected a priori based on existing literature and their theoretical relevance to adolescent health behaviors and psychosocial outcomes. Nevertheless, residual confounding cannot be ruled out. Variables such as parental education, household income, peer influence, and school-level environmental factors were not available and may have influenced the observed associations.

Future research should employ longitudinal and intervention-based designs using objective measures of lifestyle behaviors, incorporate a broader range of psychosocial factors, and adopt cross-cultural and mixed-methods approaches to clarify causal pathways and inform the development of effective, culturally sensitive interventions aimed at enhancing adolescent self-esteem and mental health.

## 5. Conclusions

This pilot study adds to the emerging literature emphasizing the relevance of healthy lifestyle behaviors for adolescents’ psychological well-being. By examining multiple lifestyle components simultaneously (physical activity, dietary patterns, and body mass index), the findings offer evidence that self-esteem in adolescence is closely linked to everyday health-related behaviors. Within this integrated framework, physical activity appeared as the strongest positive correlate of self-esteem, followed by balanced nutritional habits, whereas unhealthy eating patterns and higher BMI were inversely related to self-esteem. These observations highlight the close interplay between physical and psychological functioning during adolescence, a developmental stage marked by rapid change and increased vulnerability.

Although the present sample was drawn from a single urban area in Romania using convenience sampling and cannot be considered representative of adolescents across Eastern Europe, the study provides exploratory insights grounded in this specific sociocultural context. The alignment of the observed associations with findings from international research suggests that the links between lifestyle behaviors and self-esteem may reflect broadly shared mechanisms, even though their magnitude and expression are likely influenced by cultural and environmental factors.

## Figures and Tables

**Table 1:** Structure of the lifestyle and mental health questionnaire: domains, scoring, descriptive statistics, and internal consistency (Cronbach’s α).

Domain		Number of Items	Score Range	Mean ± SD	Cronbach’s α
Physical Activity		3	3–15	3.8 ± 1.9	0.83
Nutrition	Healthy	7	7–35	8.4 ± 2.6	0.81
	Unhealthy	7	7–35	5.7 ± 2.3	0.78
Mental Health & Body Image		10	10–50	19.6 ± 4.8	0.84
Total Lifestyle & Mental Health Score		27	27–135	31.9 ± 6.2	–

**Table 2:** Participants’ Baseline Characteristics.

Age (Years)	*n* (♂)	*n* (♀)	*n* (Total)
14	4	3	7
15	5	6	11
16	7	11	18
17	6	11	17
18	23	37	60
Total	45	68	113
**Dietary Pattern**			**% (Total)**
No specific diet	29	38	59.3%
Balanced diet	10	15	22.1%
Low-carbohydrate diet	3	5	7.1%
Portion-reduction diet	6	2	7.1%
Gluten-free diet	3	2	4.4%
Total	44	69	100%

Note: Diet types were self-identified by participants in response to a single self-report item asking them to choose the option that best matched their current eating behavior. No participants reported following a vegetarian diet.

**Table 3:** Descriptive statistics for self-esteem and lifestyle indices.

Variable	Mean	SD
Rosenberg Self-Esteem	28.68	2.98
Balanced Nutrition	3.11	0.60
Unhealthy Nutrition	2.87	0.44
Physical Activity & Supplements	3.13	0.61

**Table 4:** Pearson correlations between lifestyle indices and self-esteem.

Lifestyle Indices	r *	*p*
Balanced Nutrition	0.469	<0.001
Unhealthy Nutrition	−0.334	<0.001
Physical Activity & Supplements	0.836	<0.001
BMI	−0.646	<0.001

* Pearson r coefficients, two-tailed tests; *n* = 113.

**Table 5:** Regression predicting self-esteem from lifestyle variables.

Predictor	β	*p*
Balanced Nutrition	1.504	<0.001
Unhealthy Nutrition	−0.700	0.014
Physical Activity & Supplements	4.368	<0.001

Model R^2^ = 0.773; dependent variable = Rosenberg self-esteem total; *n* = 113.

## Data Availability

The original contributions presented in this study are included in the article material. Further inquiries can be directed to the corresponding authors.

## References

[1] Trzesniewski K.H., Donnellan M.B., Moffitt T.E., Robins R.W., Poulton R., Caspi A. (2006). Low Self-Esteem during Adolescence Predicts Poor Health, Criminal Behavior, and Limited Economic Prospects during Adulthood. Dev. Psychol..

[2] Krobtrakulchai T., Puranachaikere T., Atsariyasing W., Viravan N., Thongchoi K., Prommin P. (2024). Enhancing Adolescent Self-Esteem: A Pilot Randomized Controlled Trial of the Online Mindfulness-Based Intervention Program (MBSI Online). Siriraj Med. J..

[3] Hoffman A.J., Schacter H.L. (2024). The Promise of an Identity-Based Self-Affirmation Intervention in Protecting against Self-Esteem Declines at the High School Transition. Dev. Psychol..

[4] Zhao L., Ngai S.S. (2022). Perceived Discrimination at School and Developmental Outcomes among Bai Adolescents: The Mediating Roles of Self-Esteem and Ethnic Identity. Int. J. Environ. Res. Public Health.

[5] Tian L., Liu L., Shan N. (2018). Parent–Child Relationships and Resilience Among Chinese Adolescents: The Mediating Role of Self-Esteem. Front. Psychol..

[6] Bogaerts A., Claes L., Raymaekers K., Buelens T., Bastiaens T., Luyckx K. (2023). Trajectories of Adaptive and Disturbed Identity Dimensions in Adolescence: Developmental Associations with Self-Esteem, Resilience, Symptoms of Depression, and Borderline Personality Disorder Features. Front. Psychiatry.

[7] Orth U., Robins R.W. (2022). Is High Self-Esteem Beneficial? Revisiting a Classic Question. Am. Psychol..

[8] Kim J., Rajaguru V. (2025). Determinants of Psychosocial and Mental Health Risks of Multicultural Adolescents: A Multicultural Adolescents Panel Study 2023. Healthcare.

[9] Sowislo J.F., Orth U. (2013). Does Low Self-Esteem Predict Depression and Anxiety? A Meta-Analysis of Longitudinal Studies. Psychol. Bull..

[10] Orth U., Robins R.W., Meier L.L. (2009). Disentangling the Effects of Low Self-Esteem and Stressful Events on Depression: Findings from Three Longitudinal Studies. J. Personal. Soc. Psychol..

[11] McClure A.C., Tanski S.E., Kingsbury J., Gerrard M., Sargent J.D. (2010). Characteristics Associated With Low Self-Esteem Among US Adolescents. Acad. Pediatr..

[12] Keane L., Loades M. (2017). Review: Low Self-esteem and Internalizing Disorders in Young People—A Systematic Review. Child Adolesc. Ment. Health.

[13] Solberg O.M., Cortés-García L., Rodríguez-Cano R., Wichstrøm L., von Soest T. (2025). From adolescence to midlife: Within-person associations between self-esteem and internalizing symptoms across 28 years. J. Affect. Disord..

[14] Salcudean A., Trusculescu L.M., Popovici R.A., Serb N., Pasca C., Bodo C.R., Craciun R.E., Olariu I. (2024). Dental anxiety—A psychosocial cause affecting the quality of life—A systematic review. Rom. J. Oral Rehabil..

[15] Chong-Wen W., Sha-Sha L., Xu E. (2022). Mediating Effects of Self-Esteem on the Relationship between Mindful Parenting and Social Anxiety Level in Chinese Adolescents. Medicine.

[16] De Jong P.J., Sportel B.E., De Hullu E., Nauta M.H. (2012). Co-Occurrence of Social Anxiety and Depression Symptoms in Adolescence: Differential Links with Implicit and Explicit Self-Esteem?. Psychol. Med..

[17] De Arellano A., Neger E.N., Rother Y., Bodalski E., Shi D., Flory K. (2023). Students’ Ratings of School Climate as a Moderator between Self-esteem and Internalizing Symptoms in a Community-based High School Population. Psychol. Sch..

[18] Sampasa-Kanyinga H., Lien A., Hamilton H.A., Chaput J.-P. (2022). Canadian 24-h Movement Guidelines, Life Stress, and Self-Esteem Among Adolescents. Front. Public Health.

[19] Knox E., Muros J.J. (2017). Association of Lifestyle Behaviours with Self-Esteem through Health-Related Quality of Life in Spanish Adolescents. Eur. J. Pediatr..

[20] Kriaučionienė V., Gajewska D., Raskilienė A., Myszkowska-Ryciak J., Ponichter J., Paulauskienė L., Petkevičienė J. (2024). Associations Between Body Appreciation, Body Weight, Lifestyle Factors and Subjective Health Among Bachelor Students in Lithuania and Poland: Cross-Sectional Study. Nutrients.

[21] Wang C.-H., Lopez-Fernandez O. (2019). Shades of Foods: Prevalence and Correlates of Food Addiction. Aloma.

[22] Reyes-Molina D., Alonso-Cabrera J., Nazar G., Parra-Rizo M.A., Zapata-Lamana R., Sanhueza-Campos C., Cigarroa I. (2022). Association between the Physical Activity Behavioral Profile and Sedentary Time with Subjective Well-Being and Mental Health in Chilean University Students during the COVID-19 Pandemic. Int. J. Environ. Res. Public Health.

[23] Ruiz-Ranz E., Asín-Izquierdo I. (2025). Physical Activity, Exercise, and Mental Health of Healthy Adolescents: A Review of the Last 5 Years. Sports Med. Health Sci..

[24] Teychenne M., Costigan S.A., Parker K. (2015). The Association between Sedentary Behaviour and Risk of Anxiety: A Systematic Review. BMC Public Health.

[25] Hoare E., Milton K., Foster C., Allender S. (2016). The Associations between Sedentary Behaviour and Mental Health among Adolescents: A Systematic Review. Int. J. Behav. Nutr. Phys. Act..

[26] Runacres A., Mackintosh K.A., Knight R.L., Sheeran L., Thatcher R., Shelley J., McNarry M.A. (2021). Impact of the COVID-19 Pandemic on Sedentary Time and Behaviour in Children and Adults: A Systematic Review and Meta-Analysis. Int. J. Environ. Res. Public Health.

[27] Răchită A.I.C., Strete G.E., Sălcudean A., Ghiga D.V., Rădulescu F., Călinescu M., Nan A.G., Sasu R.D., Suciu L.M., Mărginean C. (2023). Prevalence and Risk Factors of Depression and Anxiety among Women in the Last Trimester of Pregnancy: A Cross-Sectional Study. Medicina.

[28] Laurier C., Pascuzzo K., Jubinville V., Lemieux A. (2024). Physical activity and its benefits on adolescents’ mental health through self-esteem. Front. Child. Adolesc. Psychiatry.

[29] Tilincă M.C., Antal C., Balint A., Sălcudean A., Varga A. (2023). The newest therapeutically approach of “diabesity” using GLP-1 RA molecules: Impact of the oral formulation. Farmacia.

[30] Salcudean A., Osz B.E., Bodo C.R., Muntean D.L. (2024). Serum serotonin level can be used as a predictive marker for depression in patients with type 2 diabetes mellitus. True or false?. Cancer.

[31] Almoraie N.M., Alothmani N.M., Alomari W.D., Al-amoudi A.H. (2025). Addressing Nutritional Issues and Eating Behaviours among University Students: A Narrative Review. Nutr. Res. Rev..

[32] Monserrat-Hernández M., Checa-Olmos J.C., Arjona-Garrido Á., López-Liria R., Rocamora-Pérez P. (2023). Academic Stress in University Students: The Role of Physical Exercise and Nutrition. Healthcare.

[33] Kok T., Wiriantono V., Aditama L., Bakhriansyah J. (2023). The Factors Affecting The Occurrence of Obesity in College Students. Unnes J. Public Health.

[34] De Matos A.P., Rodrigues P.R.M., Fonseca L.B., Ferreira M.G., Muraro A.P. (2021). Prevalence of Disordered Eating Behaviors and Associated Factors in Brazilian University Students. Nutr. Health.

[35] Jiménez-Morcillo J., Ramos-Campo D.J., Rodríguez-Besteiro S., Clemente-Suárez V.J. (2024). The Association of Body Image Perceptions with Behavioral and Health Outcomes among Young Adults. Nutrients.

[36] WHO Global School-based Student Health Survey (GSHS). World Health Organization. https://www.who.int/teams/noncommunicable-diseases/surveillance/systems-tools/global-school-based-student-health-survey.

[37] Roberts C., Freeman J., Samdal O., Schnohr C.W., de Looze M.E., Nic Gabhainn S., Iannotti R., Rasmussen M. (2009). International HBSC Study Group. The Health Behaviour in School-aged Children (HBSC) study: Methodological developments and current tensions. Int. J. Public Health.

[38] Kotnik K.Z., Jurak G., Starc G., Golja P. (2017). Faster, stronger, healthier: Adolescent-stated reasons for dietary supplementation. J. Nutr. Educ. Behav..

[39] Carneiro J.R., Confortin S.C., Viola P.C.D.A.F., Silva A.A.M.D. (2023). Is There an Association between Food Consumption According to the Degree of Processing and Body Image (Dis)Satisfaction in Adolescents?. Nutrients.

[40] Swami V., Horne G., Furnham A. (2021). COVID-19-Related Stress and Anxiety Are Associated with Negative Body Image in Adults from the United Kingdom. Personal. Individ. Differ..

[41] Shi Y., Tang Z., Gan Z., Hu M., Liu Y. (2023). Association Between Family Atmosphere and Internet Addiction Among Adolescents: The Mediating Role of Self-Esteem and Negative Emotions. Int. J. Public Health.

[42] Choi Y., Choi S.-H., Yun J.-Y., Lim J.-A., Kwon Y., Lee H.Y., Jang J.H. (2019). The Relationship between Levels of Self-Esteem and the Development of Depression in Young Adults with Mild Depressive Symptoms. Medicine.

[43] Buicu G.E., Gabos Grecu M., Salcudean A., Gabos Grecu I., Marinescu C., Nirestean A., Turliuc S., Hadareanu V., Udristoiu I. (2017). Evaluation of Elder Physical Abuse. Eur. Psychiatr..

